# Frank W. Abbott, M. D.

**Published:** 1894-04

**Authors:** Frank W. Abbott

**Affiliations:** Chairman Training School Committee, B. G. H.; Buffalo


### Correspondence.

*To the Editor of the Buffalo Medical and Surgical Journal :*

SIR—In the current number of your journal, you very justly commend the action of Mrs. Chace, of Providence, in devising money to build a Nurses' Home for the local training school for nurses, but we need not go so far afield to find an even more commendable example.

In 1890, the Buffalo General Hospital Training School for nurses entered an elegant and completely equipped nurses' home, the donation of a lady, who gave us the benefit of her practical wisdom in the selection of a plan, and in supervising the details of construction and furnishing, as well as the money wherewith to build and furnish. Last summer, learning that the nurses were considering how they might obtain a piano, she ordered an upright Steinway, which now adorns the parlor, and is the source of daily delight and entertainment.

Is it not better to give to a training school while living and enjoy the satisfaction of seeing the improved health and increased happiness of these hard-working young women, than to depend on the uncertainties of a bequest ?

Upon a brass tablet in the main hall is the following inscription :

" As a grateful recognition of the generosity of Sarah H. Gates, in building and furnishing the Nurses' Home, this tablet is placed by the nurses of the Buffalo General Hospital, in the year of Our Lord, 1892."

And thou shalt be blessed, for they cannot recompense thee, for thou shalt be recompensed at the resurrection of the just.—St. Luke xiv., 14.

FRANK W. ABBOTT, M. D.,
*Chairman Training School Committee, B. G. H.*

BUFFALO, March 9, 1894.

